# A monitoring and feedback tool embedded in a counselling protocol to increase physical activity of patients with COPD or type 2 diabetes in primary care: study protocol of a three-arm cluster randomised controlled trial

**DOI:** 10.1186/1471-2296-15-93

**Published:** 2014-05-12

**Authors:** Renée Verwey, Sanne van der Weegen, Marieke Spreeuwenberg, Huibert Tange, Trudy van der Weijden, Luc de Witte

**Affiliations:** 1 School for Public Health and Primary Care (CAPHRI), Maastricht University, Maastricht, the Netherlands; 2 Research Centre Technology in Care, Zuyd University of Applied Sciences, Heerlen, the Netherlands

**Keywords:** Physical activity, Self-management support, Primary care nursing, Remote sensing technology

## Abstract

**Background:**

Physical activity is important for a healthy lifestyle. Although physical activity can delay complications and decrease the burden of the disease, the level of activity of patients with chronic obstructive pulmonary disease (COPD) or type 2 Diabetes Mellitus (DM2) is often far from optimal. To stimulate physical activity, a monitoring and feedback tool, consisting of an accelerometer linked to a smart phone and webserver (*It’s LiFe!* tool), and a counselling protocol for practice nurses in primary care was developed (the Self-management Support Program). The main objective of this study is to measure the longitudinal effects of this counselling protocol and the added value of using the tool.

**Methods/Design:**

This three-armed cluster randomised controlled trial with 120 participants with COPD and 120 participants with DM2 (aged 40–70), compares the counselling protocol with and without the use of the tool (group 1 and 2) with usual care (group 3). Recruitment takes place at GP practices in the southern regions of the Netherlands. Randomisation takes place at the practice level. The intended sample (three arms of 8 practices) powers the study to detect a 10-minute difference of moderate and intense physical activity per day between groups 1 and 3. Participants in the intervention groups have to visit the practice nurse 3–4 times for physical activity counselling, in a 4-6-month period. Specific activity goals tailored to the individual patient's preferences and needs will be set. In addition, participants in group 1 will be instructed to use the tool in daily life. The primary outcome, physical activity, will be measured in all groups with a physical activity monitor (PAM). Secondary outcomes are quality of life, general - and exercise - self-efficacy, and health status. Follow-up will take place after 6 and 9 months. Separately, a process evaluation will be conducted to explore reasons for trial non-participation, and the intervention’s acceptability for participating patients and nurses.

**Discussion:**

Results of this study will give insight into the effects of the *It’s LiFe!* monitoring and feedback tool combined with care from a practice nurse for people with COPD or DM2 on physical activity.

**Trial registration:**

ClinicalTrials.gov: NCT01867970

## Background

Because increased physical activity (PA) has positive effects on prognosis and quality of life [1,2], stimulating PA is an important element in the treatment of people with chronic diseases such as chronic obstructive pulmonary disease (COPD) or type II diabetes (DM2) [3,4]. It is, however, a challenge to adhere to guidelines for healthy exercise (at least 30 minutes of moderate activity five days a week) [5,6]. By integrating PA counselling into routine practice, primary care providers can support patients in meeting this challenge [5,7]. In the Netherlands the majority of chronically ill patients visit the family practice regularly to monitor their condition, and it is the task of the practice nurse (PN) to provide lifestyle counselling during those consultations [8,9].

The most common method of PA promotion is verbal advise, followed by print- and computer-based interventions [10]. Interventions incorporating technology that is readily accessible on a daily basis for monitoring activity levels, such as computers or mobile phones, can support care providers to coach patients in establishing behavioural changes [11]. Those interventions may facilitate long-term follow-up [12,13], and may be an effective way to provide PA counselling without increasing the time demands on primary care providers [14].

PA counselling has the potential to increase PA levels in the short term [13]. However, evidence regarding which methods of exercise promotion works best in the long term is still limited [15]. Furthermore, computer-based patient self-management programs, delivered in health-supported settings, show the potential for changing health behaviours and improving clinical outcomes, but more well designed trials are warranted to test their effectiveness [16]. Those trials should especially focus on the effects of theory-based intervention development, combined with the effect of tailored advise and feedback [17].

We therefore, developed and tested a monitoring and feedback tool called *It’s LiFe!*[18,19] and a corresponding counselling program for primary care nurses (the Self-management Support Program). The basic ideas behind this combination are: providing an objective measurement of PA via an accelerometer, collaborative goal setting and automatic feedback via an application on a smartphone combined with PA counselling by the PN. Results from a feasibility study showed that participants were positive about the tool. Regarding the effects of using the tool, a positive trend was seen: the mean level of PA increased by more than 10 minutes per day and patients reported a higher quality of life [20].

This paper describes the study protocol of a three-armed cluster randomised controlled trial with 120 participants with COPD and 120 participants with DM2 (aged 40–70), comparing the Self-management Support Program with and without the use of the tool (group 1 and 2) with usual care (group 3).

### Objectives and hypotheses

The objective of this randomised controlled trial is to evaluate the longitudinal effects of the *It’s LiFe!* tool embedded in a Self-management Support Program (SSP) on 40–70 years old patients with COPD and DM2 in primary care. The primary outcome measure is PA in daily life. Secondary outcome measures are self-efficacy, quality of life and health status. The main difference that is evaluated is between the whole intervention and usual care. Additionally, the isolated effect of the tool is evaluated. Apart from the effect evaluation, a process evaluation will be performed, aimed at getting insight into the adherence to the intervention and the acceptance of the intervention by participating patients and PNs.

The main hypothesis is that the whole intervention will increase PA on a moderate level by at least 10 minutes per day, over a four to six-month period, and to maintain this increase over three months.

## Methods/design

This paper was written according to the CONSORT 2010 statement: extension to cluster randomised trials [21].

### Study design

**Figure 1:**
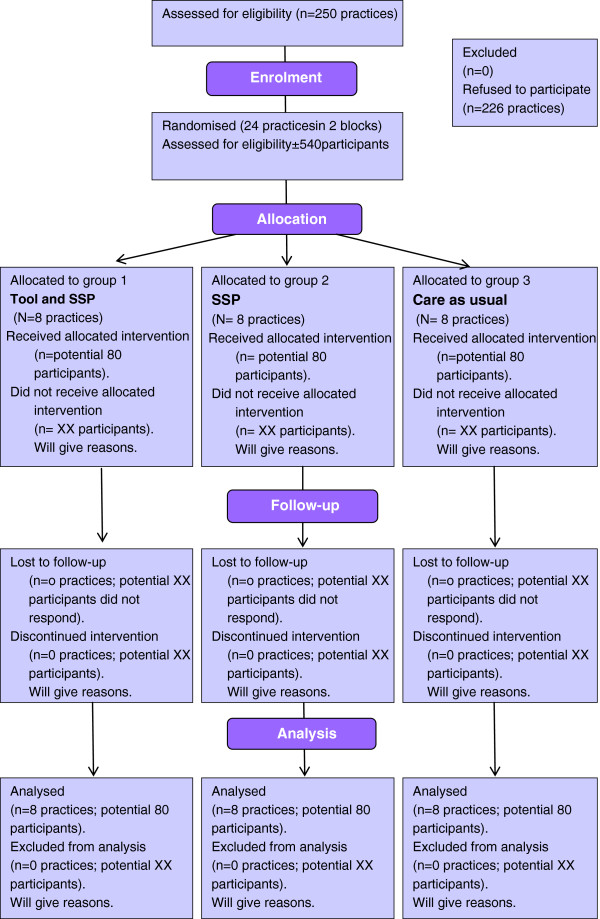
CONSORT flowchart trial design; potential flow of participants.

The study is designed as a cluster randomised controlled trial with GP practices as the unit of randomisation. To compare the whole intervention with both usual care and SSP only (to isolate the effect of the tool), the trial has three arms: the use of a monitoring and feedback tool embedded in the SSP (group 1), the SSP without the tool (group 2), and usual care (group 3). The CONSORT flowchart (Figure 1) summarises the trial design. The population consists of 120 participants with COPD and 120 participants with DM2 from 24 GP practices. Each practice provides 5 COPD patients and 5 DM2 patients, which makes a total of 40 patients with COPD and 40 patients with DM2 from 8 practices per trial arm.

### Eligibility

Participants between 40 and 70 years old are eligible when they are diagnosed with COPD or DM2, are treated in primary care, and in the opinion of the PN, do not comply with the Dutch Norm for Healthy Exercise [22]. Additional inclusion criteria for the DM2 patients are a BMI > 25 and for the COPD patients: a clinical diagnosis of COPD according to the GOLD-criteria stage 1–3, being at least six weeks respiratory stable and on a stable drug regimen. Furthermore, patients should have access to a computer with an internet connection.

Exclusions are patients with coexisting medical conditions with a low survival rate, severe psychiatric illness or chronic disorders or diseases that seriously influence the ability to be physically active and those being primarily treated by a medical specialist or participating in another PA intervention, as well as patients with insufficient mastery of the Dutch language.

### Recruitment

#### Recruitment of practices

GP practices located in southern regions of the Netherlands will be approached by an invitation letter, by telephone and personal contact with GP’s, practice managers, and PNs, to invite them to participate in the study, until a maximum of 24 practices is reached. On the basis of the number of patients with DM2 treated per practice, the practices will be categorised into small (<90), medium (90–190), large (190–390) and extra-large (>390).

#### Recruitment of participants

To recruit participants for the study, PNs will identify 20–32 eligible patients per practice, who fulfil the inclusion criteria. This will be done before the randomisation of the practices. When the PN considers a patient eligible for participation, the nurse will send a recruitment letter to the patient with general information about all groups. After the randomisation, the PN will call those patients to give specific information about the group in which the practice is allocated and to ask patients if they want to participate; non-responders will be asked for their reasons not to participate. Each general practice will be instructed to include 10–14 participants, with an equal distribution of COPD and DM2 patients. When the patient decides to participate, he or she will receive an informational letter and informed consent form.

### Randomisation procedure

A total of 24 practices will be randomly allocated into the three groups in two blocks of twelve practices. Before randomisation, the practices will be pre-stratified into four strata based on the size of the practice. The practices will be stratified into groups of 3 per size and randomised by an independent person into either one of the two intervention groups or the control group by numbering sealed envelopes which contain the names of the practices.

As they have to contact participating nurses to inform them about the relevant intervention, the executing researchers (SvdW & RV) will be aware of which practices are in which group. Patient data will be analysed anonymously, without any recognition of names or practices. An independent person will store the coding key. All cleaning and processing of data will be carried out on the whole database (i.e., all three groups). The group and practice variable will only be revealed at the end of the study.

### Intervention

**Figure 2:**
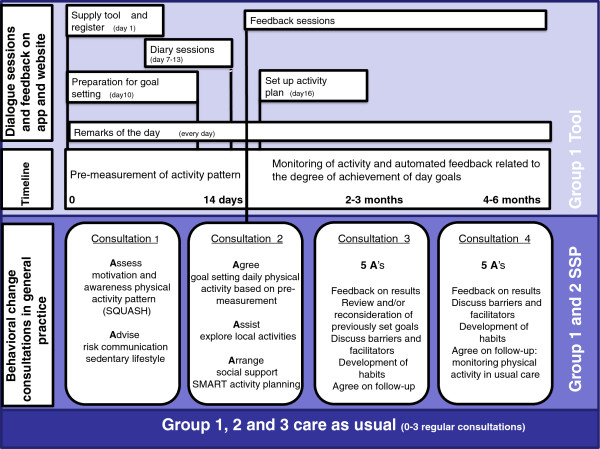
Interventions RCT It’s LiFe!

The different components of the interventions are summarised in Figure 2.

The interventions have been designed in a user-centred manner; two patient representatives, from the Netherlands Asthma Foundation and the Dutch Diabetes Association, participated in the research group to provide feedback on every aspect of the project.

#### The tool (Group 1)

**Figure 3:**
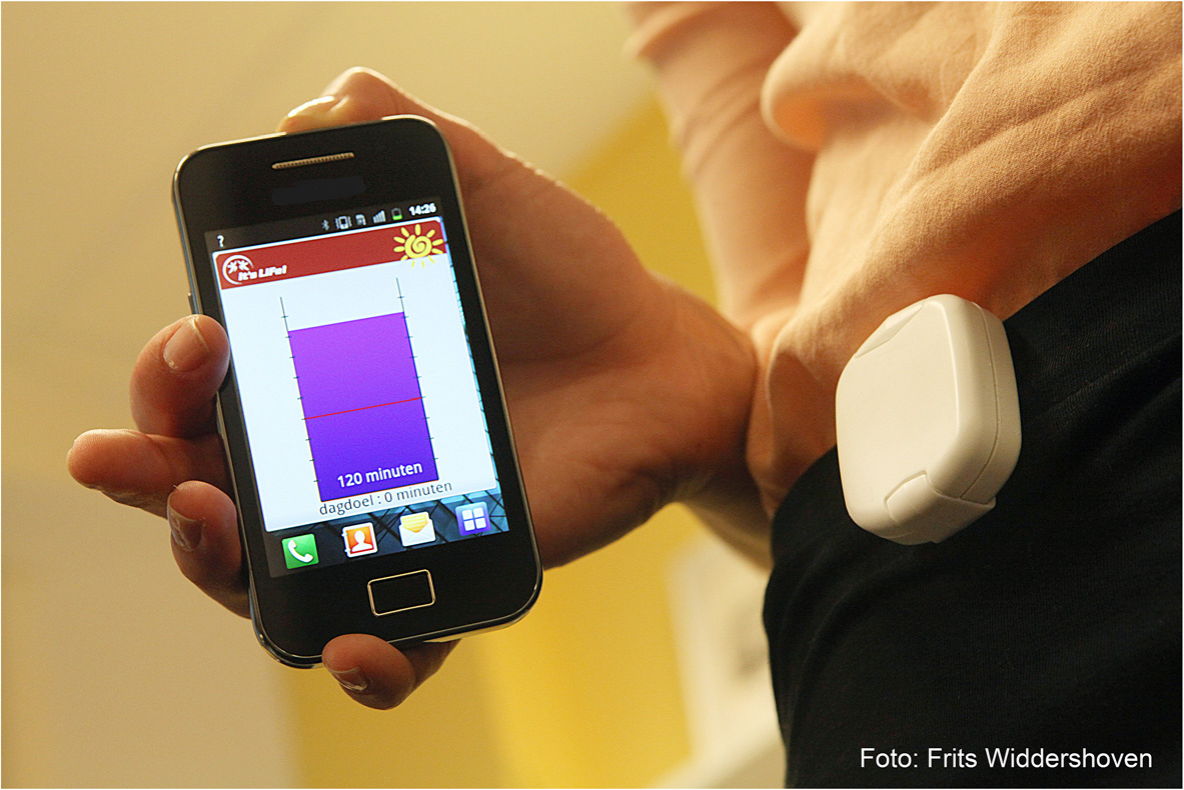
**The *It’s LiFe! *** **tool.**

The *It’s LiFe!* tool (Figure 3) consists of an accelerometer, a smartphone app, and a server/web application. Participants receive personalised feedback on the smartphone concerning their amount of activity in relation to an activity goal, which is set in dialog with their PN [18] after a two week pre-measurement period. Nurses can monitor patients’ PA via a secure website [19].

The use of the tool starts when the participant is registered on the server by the PN. The server has two portals, one for care providers (*It’s LiFe!* monitor) and one for patients (*It’s LiFe!* online). The PN creates an account for the participant and then the log-in name and password are sent by email. At home, the participant has to complete a short questionnaire online (a dialog session) concerning PA preferences and has to log in on the phone. Daily at 1 a.m. the smartphone automatically connects to the server to upload the PA data from the previous day. There is a pre-measurement period of 14 days. Participants can enter “remarks of the day” whenever they want, such as comments about being sick or having forgotten to wear the meter. In the second week, they receive dialog sessions about the enjoyment and exertion of performed activities. Furthermore, participants receive two sessions from the server concerning barriers and facilitators and activity planning based on the Physician-based Assessment and Counselling for Exercise intervention (PACE) [23], with the aim of modifying factors known to influence PA, such as social support and self-efficacy. After two weeks, together the patient and nurse set a goal in minutes of activity per day, which is entered into the system by the nurse. Based on the PA data related to this goal, participants receive feedback sessions. There are several types of messages (e.g., tips, encouragement, positive trend, reward, barriers, facilitators and the suggestion to adjust goals). Participants will get such messages when they reach their goal after 3, 5 and 14 days or when they do not reach their target after 3, 5 and 14 days. In some cases, the goals have to be reached 100 % and others are based on 80 % achievement. All messages are written in a positive tone, e.g., ‘Good that you still try to be more active. We can see that it is hard to reach your daily target. If you want to adjust your goal, contact your care provider or click here’.

#### Instruction tool

The PNs in group 1 practices will receive a personal account for the monitor, a manual and the researchers (SvdW & RV) will instruct PNs on how to use the system. Researchers (SvdW & RV) will also advise the nurses to try out the tool themselves and to sign up as a patient in the system to get familiar with it. In addition to a manual, there are several short instructional films available on a special website (see http://www.maastrichtuniversity.nl/web/show/id=6637066/langid=43); the films cover a variety of topics, for example, how to log on to the app and how to respond to a session. For technical questions about the use of the tool, participating patients and PNs are able to contact a helpdesk during working hours.

#### The Self-management Support Program (Groups 1 and 2)

The intervention in group 1 consists of the use of the tool in daily living, intertwined with consultations with the PN – the Self-Management Support Program (SSP). The intervention in group 2 consists of this program without the use of the tool. The program is based on the Five A’s model (Assess, Advise, Agree, Assist, Arrange), a counselling protocol to support self-management in a primary care setting [24,25].

This program consists of four consultations with the PN: in the first week, after 2 weeks, after 8–12 weeks and after 16–24 weeks. Before the consultations, the participants receive an informational booklet about the course of the intervention containing the Short Questionnaire to Assess Health-enhancing PA (SQUASH) [26] and a list of locally organised PA options. The duration of the consultations is 20 minutes, or a 10-minute extension of a regular consultation. In the first consultation, the PN will try to increase awareness of the PA pattern of the patient, and inform the patient about the health risks related to a sedentary lifestyle. The patient and the PN will get an idea about the PA level of the patient by discussing the previously completed SQUASH questionnaire. Furthermore, the patient gets a leaflet with disease specific information related to PA [27,28].

During the second consultation, a goal will be set regarding physical activity in minutes per day, based on the results of the measurements of the first two weeks (pre-measurement). The pre-measurement in group 1 is an objective measurement based on the tool, in group 2 this is a subjective measure achieved by asking participants to keep a PA diary. The results of the pre-measurement of group 1 are visible for the nurse on the monitor portal of the *It’s LiFe!* server. In both intervention groups, the nurse will encourage the patient to focus on goals that fit the patient’s preferences and to set up a Specific, Measureable, Attainable, Realistic, and Timely (SMART) plan to reach personal goals, and the nurse will inform the participant about locally organised exercise opportunities.

In the third consultation, possibly by mail or telephone, the nurse will discuss the results, barriers and facilitators related to PA. In the last consultation, the nurse will discuss the results, behaviour change(s) and habits with the participant. The proposed behaviour change counselling techniques have been classified according to Abraham and Michie’s taxonomy as listed in Table 1[29].

**Table 1:** **Details of the tool and the PA counselling consultations and proposed Behavioural Change Techniques**[29]

**Condition 1: Tool**		
**Proposed Behavioural Change Techniques (BCT)**		**Number according to BCT Taxonomy Abraham and Michie**
**Tool widget (continuous)**	Prompt specific goal setting	10
	Provide feedback on performance	13
	Prompt review of behavioural goals	11
**Tool sessions**	Provide general encouragement	6
	Provide general information	1
	Provide information on consequences	2
	Prompt intention formation	4
	Plan social support/social change	20
	Prompt barrier identification	5
**Condition 1 and 2: Self-management Support Programme**		
**Consultation 1**	Provide general information	1
	Motivational interviewing	24
	Provide general encouragement	6
	Provide information on consequences	2
	Prompt intention formation	4
**Consultation 2**	Provide general encouragement	6
	Motivational interviewing	24
	Prompt specific goal setting	10
	Plan social support/social change	20
**Consultation 3**	Provide general encouragement	6
	Provide feedback on performance	13
	Motivational interviewing	24
	Prompt review of behavioural goals	11
	Prompt barrier identification	5
	Relapse prevention	23
**Consultation 4**	Provide general encouragement	6
	Provide feedback on performance	13
	Motivational interviewing	24
	Prompt review of behavioural goals	11
	Prompt barrier identification	5
	Relapse prevention	23

#### Instruction for SSP

Informational booklets are produced, focusing on PA behaviour change, with an explanation and a timeline of the intervention. Before the start of the intervention, these booklets will be sent to participants.

The nurses in group 1 and 2 practices will receive a personal instruction at their workplace; these instructions will also be available as an online web lecture. The nurses will receive an information file with detailed instruction charts for the course of each consultation, and an explanation of the intended counselling techniques.

#### Care as usual (group 3)

Care as usual (for all three groups) consists of regular consultations with the PN (COPD patients have 1–2 consultations and DM2 patients have 4 consultations per year). Participants in the usual care group will not be offered any programme besides usual contacts with the GP and PN.

### Data collection

All participants are asked (by a letter from the researchers) to wear the PAM and complete questionnaires at three different time points; namely at baseline (t0), at the end of the intervention after 4–6 months (t1), and at follow-up, 3 months after the end of the intervention (t2). Measurements and time points are summarised in Table 2.

**Table 2:** Measurements and time points

**Concept (questionnaires)**	**Intervention groups**			**Control group**		
	**t0**	**t1**	**t2**	**t0**	**t1**	**t2**
Demographic variables	x			x		
Physical activity (PAM)	x	x	x	x	x	x
Quality of life (SF 36)	x	x	x	x	x	x
General Self-Efficacy (GSS)	x	x	x	x	x	x
Exercise Self-Efficacy (ESS)	x	x	x	x	x	x
Health status (DSC-R or CRQ-SAS)	x	x	x	x	x	x
Process evaluation		x				

PAM: Personal Activity Monitor.

DSC-R: Diabetes Symptom Checklist-Revised.

CRQ-SAS: Chronic Respiratory Questionnaire-Self-Administered Standardised.

T0 - baseline.

T1 - after 4–6 months (end of intervention).

T2 - after 9 months (post intervention).

### Outcome parameters

#### Primary outcome measure

##### Physical activity

PA will be measured with the Personal Activity Monitor (PAM AM300) [30]. The PAM is a small tri-axial accelerometer that can be easily attached to a belt and is worn on the hip. The PAM registers all hip movements that are made during a day. Via a docking station, and connection to the internet, the PAM scores and data of minutes a day in a sedentary category (<1.8 METS), a living category (1.8-3 METS), a moderate category (3–6 METS), and a vigorous category (>6 METS) will be uploaded [30]. The number of minutes of PA in the moderate and vigorous category (>3 METS) will be considered as the primary outcome measure. We will also report about the number of minutes of PA in the living, moderate and vigorous category >1.8 METS. These measures indicate all types of activity during the day. The possibility for the users of noticing their activity scores on the PAM will be deactivated; the displays will only show a digital clock. Participants will be asked to wear the PAM during 8 consecutive days for more than 12 hours a day. They will be asked to register the days and times that they wear the PAM; activities that are difficult to measure (swimming, cycling and strength training) will be recorded on a paper log. A measurement will be considered valid if the wear time is > 8 hours per day and if there is data of > 5 days.

#### Secondary outcome measures

##### Quality of life

To measure the quality of life the SF-36 will be used [31,32]. The SF-36 consists of 36 items, organised into 8 subscales, including vitality, physical functioning, body pain, general health perceptions, emotional role functioning, social role functioning, and mental health. A higher score indicates a better quality of life.

##### Self-efficacy

An important mediator of PA behaviour is self-efficacy; therefore this will be measured with two different questionnaires. The 10-item General Self-efficacy Scale (GSS) is designed to assess optimistic self-beliefs to cope with a variety of difficult demands in life, scores for each item range from 1 (totally disagree) to 4 (totally agree) [33]. The Exercise Self-efficacy Scale (ESS) describes 18 situations during which it could be difficult to adhere to an exercise routine, for example ‘without support from family and friends’. Participants are asked to rate their degree of confidence to continue with regular exercise in the listed situations. The ESS uses a 100-point scale for each item, ranging from 0 ‘I cannot do this at all’ to 100 ‘I am certain that I can do it’, with higher scores reflecting higher levels of exercise self-efficacy [34-36].

#### Additional measures

##### Health status

Personal reported health status will be measured by two disease specific questionnaires, the Diabetes Symptom checklist-revised (DSC-R) for participants with DM2 and the Chronic Respiratory Questionnaire (CRQ) for participants with COPD.

DSC-R consists of 34 items and 8 sub-dimensions; hyperglycaemia, hypoglycaemia, psychological – cognitive, psychological – fatigue, cardiovascular, neurological –pain, neurological – sensoric and ophtalmological. On the DSC-R, patients indicate for each of the 34 listed symptoms whether or not they suffered from it in the last month. If they did experience the symptom, patients rate the perceived burden on a scale from 1 (not at all) to 5 (extremely) [37-39].

The Chronic Respiratory Questionnaire (CRQ-SAS) consists of 20 items across four dimensions: dyspnoea, fatigue, emotional function, and mastery (the patient’s feeling of control over their disease). The dyspnoea portion is individualised for each patient: the person is asked to select the 5 activities associated with breathlessness that they perform frequently and are most important to them. Dyspnoea items can be selected from a list of 26 suggested items or may be written in by the patients. Items are scored from 1 (most severe) to 7 (no impairment) [40,41].

##### Process evaluation

Because of the expected wide range of differences in the performance of the intervention by the PNs and in the adherence of patients in using the tool, a process evaluation is necessary [42,43]. The purpose of the process evaluation is to examine the context, implementation and receipt of the intervention. The evaluation consists of registration forms, a process evaluation questionnaire for participants in the intervention groups at t1, interviews by telephone with the PNs responsible for the study and focus groups with PNs at the end of the study. During the interviews, information is gathered about the inclusion of participants, the course of the consultations, the education and motivation of the PNs, experienced motivation and treatment possibilities of the participants and the perceived effect of the intervention. Time spent on the intervention is recorded on registration forms. In the questionnaires, participants in both groups and the PNs are asked about their experiences with the SSP and the tool. In Additional file 1 all process evaluation components, operationalisation, and measurements are summarised, according to the framework of Saunders [44].

##### Sample size and power calculation

For this study, 240 patients are required, with a minimum of 80 participants per group. Based on a validation study, we assume that the PA level of participants is an average of 24 minutes with a range of 14.6 minutes. A mean difference between group 1 and group 3 of ten minutes (42 %) of moderate to vigorous PA spent per day will be seen as clinically relevant. While assuming an intra-class correlation of 10 % based on practice, to account for the dependency of the data, with a power of 80 % and a significance level of 0.05, a total of 72 patients over 8 general practices are required in each group. Because a drop-out rate of 10 % is expected, practices will be asked to include 8–14 patients per practice in each subgroup, depending on the size of the practice.

### Planned statistical analyses

#### Descriptive statistics

Demographic data (e.g., age, gender, disease, co-morbidities) will be described for the total group and for the subgroups separately. Continuous variables will be denoted with means, standard deviations, and medians. Categorical variables will be denoted in numbers and percentages. The participants included in the 3 arms will be tested on differences between characteristics, with chi-square, ANOVA (Kruskal Wallis).

If variables differ between groups, with a p-value ≤0.10, they will be considered to be potential confounders in further analysis.

#### Data analysis for primary and secondary outcomes

An intention to treat analysis and a per protocol analysis will be conducted. For each outcome measure (all outcomes are continuous) data will be expressed as mean +/-SD. The between group comparisons will be analysed with multilevel analysis to account for the dependency of observations. We will apply a 3 level linear mixed model (time, participant, practice); the level of statistical significance will be set at 0.05 (two-tailed). Separate models (random intercept) will be set up for each outcome measure. The independent variables in each model are two dummy variables indicating the group, with the group of patients receiving usual care as the reference category and two dummy variables for time and their interaction effects. In addition, an extra dummy variable will be included to indicate the patient group (COPD versus DM2), to study whether the effects in COPD patients differ from the effects in patients with DM2. If needed, additional baseline variables will be included to account for possible confounding. If normality assumptions are violated, outcome variables will be log-transformed and if necessary non-parametric tests will be used. SPSS, version 19 and Mlwin, version 2.02 will be used to analyse the data.

#### Data analysis process evaluation

Quantitative data will be analysed by means of descriptive statistics. In order to identify relevant themes, qualitative data (results of open-ended interviews and focus groups) will be independently analysed by two researchers using NViVo version 9. A concurrent triangulation strategy will be applied to confirm, cross validate and corroborate the findings.

### Procedure for accounting for missing, unused and unexpected data

Accounting for missing values on items in questionnaires will be handled according to the scoring algorithms of the questionnaires. Missing variables in follow-up data will not be imputed since it has been shown that multilevel analysis is a very flexible method for handling missing data [45].

### Stopping rules

There are no formal statistical stopping rules. If a patient decides to withdraw (e.g., hospital admission), the nurse may discontinue the intervention, but all participants will be asked to complete follow-up assessments. Patients can withdraw from the study at any time.

### Ethical principles

The study protocol was approved by the research ethics committee of azM/UM, Maastricht, the Netherlands in 2013 (METC12-3-071).

## Discussion

This study fills a gap in the literature about how to improve self-management of patients with COPD or DM2 in increasing their level of PA by using technology embedded in primary care.

Post-recruitment selection bias, a well-known problem of cluster randomised controlled trials, will be partly avoided by asking the nurses to include patients and send a general invitation letter before the randomisation of the practices. But not informing the patients about the intended intervention (the randomisation outcome of their GP practice), is insuperable because patients have to be informed about the intervention before they agree to participate.

During a pragmatic trial, which aims to measure the effectiveness of an intervention in routine practice, it is important to collect process data to avoid Type III errors (evaluating an intervention that was inadequately implemented). In choosing the outcomes and measurements of the process evaluation, the potential for increased Hawthorne effects will be taken into account by minimising the contacts between researchers and participants, and by avoiding overlapping roles between researchers and PNs, for example by asking the PNs to include patients for the study, and by arranging an independent helpdesk. Patients will not be interviewed during the intervention in order to distinguish between the intervention and its evaluation.

## Conclusion

In conclusion, the need to increase the level of PA in people with COPD or DM2 is evident, in which the use of a monitoring and feedback tool embedded in a counselling protocol can play an important role. In the present three-arm cluster randomised controlled trial, we will evaluate the effectiveness of this counselling protocol and the added value of using the *It’s LiFe!* monitoring and feedback tool.

## Abbreviations

COPD: Chronic obstructive pulmonary disease; DM2: Type 2 diabetes mellitus; It’s LiFe!: Interactive tool for self-management through lifestyle feedback; GP: General practitioner; PAM: Physical activity monitor AM300; SSP: Self-management Support Program.

## Competing interests

The author(s) declare that they have no competing interests.

## Authors’ contributions

LdW, TvdW, MS, HT, SvdW and RV conceived and designed the study. SvdW and RV are collecting the data. SvdW, RV and MS will analyse the data. RV wrote the paper. All authors edited, revised and approved the final manuscript.

## Pre-publication history

The pre-publication history for this paper can be accessed here:

http://www.biomedcentral.com/1471-2296/15/93/prepub

## Supplementary Material

Additional file 1 Components, operationalisation and measurements process evaluation. Click here for file
