# Supplementary material for: A monitoring and feedback tool embedded in a counselling protocol to increase physical activity of patients with COPD or type 2 diabetes in primary care: study protocol of a three-arm cluster randomised controlled trial
Source: BMC Fam Pract. 2014 May 12;15:93. doi: 10.1186/1471-2296-15-93 (PMC4030038; doi:10.1186/1471-2296-15-93)
Supplement: Additional file 1 — Components, operationalisation and measurements process evaluation. [file 1471-2296-15-93-S1.pdf]

Component and definition	Operationalisation	Measurements								
		Dropout (call participants)	System log file Server	Helpdesk logbook	Inclusion list PNs	Evaluation sheets per consultation	(Demographic) Questionnaire participants	Questionnaire PNs	Interview PN (phone)	Focus gr PNs
**Recruitment**<br>The recruitment procedures that were used.	Characteristics of participants that were invited and that refused to participate.				x					
**Reach**<br>The proportion of the intended target population that participated in the intervention groups.	Characteristics of participants.	x				x	x			
	Number of participants that completed the program or dropped out.	x								
	Reasons for withdrawal.	x								
**Context**	Characteristics of general practices.								x	
	Characteristics of PNs.								x	
**Fidelity**<br>The extent to which each of the intervention components was delivered as planned<br>• Extent to which the SSP was delivered as planned<br>• Extent to which the tool functioned as planned	Planned preparation time and duration of the consultations.					x				
	Extent to which technical problems occurred.			x			x			
**Dose delivered**<br>The extent to which the intervention components were carried out as often and for as long as planned<br>• Regarding the SSP	Consultations and other contacts between participants and PNs (dates, time, planned and executed, within regular consultations or extra).					x				
	Extent to which the PN achieved to:<br>- Assess the physical activity level of the patient<br>- Inform the participant about the risks of a sedentary lifestyle<br>- Collaborative set goals and set up an					x	x			

	action plan with the participant<br>- Give feedback based on the physical activity goals<br>- Discuss with the participant barriers and facilitators for being active<br>- Use motivational interviewing techniques									
• Regarding the tool	Tool:<br>- Functioning of the website as intended<br>- Functioning of the app as intended		x	x						
	Functioning of the monitor as intended			x						
**Dose received (exposure)**<br>Extent of participants' active engagement in and receptiveness to the intervention<br>• Regarding the SSP	Overall opinion of the participant and the PN regarding the participant's engagement in the program.					x	x		x	
• Regarding the tool: extent to which the tool was used as intended	Instruction of the tool (use of the manual and the instruction movies)						x		x	
	Adherence towards the tool (completion sessions)		x							
	Monitoring results in between consultations							x	x	
**Dose received (satisfaction)**<br>Satisfaction of participants and PNs with the different components of the intervention	Experiences using the tool<br>- Activity monitoring<br>- Sessions<br>- Feedback messages		 x<br>x<br>x				x		x	
	Experience using the *It's LiFe!* monitor by PNs during the consultations.<br>- Set up and change goals<br>- View participants' results		x		x				x	
	How satisfied were the participants and the PNs with the program, with the tool and with the combination of both?						x	x	x	x
	How did the participants and the PNs perceive the outcomes and relevance of the intervention?						x	x	x	x

Additional file 2. Components, operationalisation and measurements of the process evaluation
